# Sex-dependent differences in water homeostasis in wild-type and V-ATPase B1-subunit deficient mice

**DOI:** 10.1371/journal.pone.0219940

**Published:** 2019-08-06

**Authors:** Anil V. Nair, Wei Yanhong, Teodor G. Paunescu, Richard Bouley, Dennis Brown

**Affiliations:** Program in Membrane Biology, Center for Systems Biology and Division of Nephrology, Massachusetts General Hospital and Harvard Medical School, Boston, MA, United States of America; University of Utah School of Medicine, UNITED STATES

## Abstract

Men tend to dehydrate more than women after prolonged exercise, possibly due to lower water intake and higher perspiration rate. Women are prone to exercise-associated hyponatremia, primarily attributed to the higher water consumption causing hypervolemia. Since aquaporin-2 (AQP2) water channels in the kidney collecting duct (CD) principal cells (PCs) are involved in maintaining water balance, we investigated their role in sex-dependent water homeostasis in wild-type (WT) C57BL/6 mice. Because CD intercalated cells (ICs) may also be involved in water balance, we also assessed the urine concentrating ability of V-ATPase B1 subunit-deficient (Atp6v1b1^-/-^) mice. Upon 12-hour water deprivation, urine osmolality increased by 59% in WT female mice and by only 28% in males. This difference was abolished in Atp6v1b1^-/-^ mice, in which dehydration induced a ~30% increase in urine osmolarity in both sexes. AQP2 levels were highest in WT females; female Atp6v1b1^-/-^ mice had substantially lower AQP2 expression than WT females, comparable to the low AQP2 levels seen in both Atp6v1b1^-/-^ and WT males. After dehydration, AQP2 relocates towards the PC apical pole, especially in the inner stripe and inner medulla, and to a greater extent in WT females than in WT males. This apparent sex-dependent concentrating advantage was absent in Atp6v1b1^-/-^ females, whose reduced AQP2 apical relocation was similar to WT males. Accordingly, female mice concentrate urine better than males upon dehydration due to increased AQP2 expression and mobilization. Moreover, our data support the involvement of ICs in water homeostasis, at least partly mediated by V-ATPase, in a sex-dependent manner.

## Introduction

Water is the largest single contributor to human body mass, ranging from 50 to 60% in an average adult. Females tend to have ~10% less water than men of similar age during adulthood. Despite this, men dehydrate more than women after prolonged exercise [1], possibly due to a slightly lower water intake in men, to an increased rate of perspiration, or a combination of both [1]. However, a third explanation could be a difference in regulated water balance mediated via aquaporin water channels in the kidney, especially AQP2. Similarly, the mechanism behind exercise-associated hyponatremia (EAH) after endurance training is not well understood. It can be caused by loss of solutes (Na^+^ and K^+^) or by retaining excess total body water content, or a combination of both. In most clinical scenarios, increased water retention in the body is the reason for EAH [2–4]. One of the major risk factors in EAH is excessive water intake, with women being more prone to developing this potentially fatal condition [1, 2, 5].

Aquaporins are a family of membrane proteins that mediate water homeostasis in the body. Four members of this family are expressed in the kidney. In the early part of the nephron, proximal tubules and thin descending limbs, AQP1 is expressed along the apical and basolateral side of the tubules and helps in reabsorbing nearly 80 percent of the total filtered water from the ultrafiltrate. AQP1 is minimally regulated and is a constitutively active channel. The remaining 20% of the water needed for fluid homeostasis is reabsorbed through AQP2 localized at the apical pole of connecting segment and collecting duct epithelial cells. Circulating vasopressin (VP) released into the blood in response to body hydration status, and acting via the vasopressin receptor 2 (V2R), is the major regulator of the AQP2 channel. In rats, males express more AQP1 than females. A strong androgen-dependent stimulation was shown to be responsible for this difference [6]. In spiny mice, there is no sex dependency in the renal expression of V2R or AQP3 [7] under basal conditions. Conversely, another study conducted in Sprague-Dawley rats showed an increased expression of V2R mRNA and protein in females compared to males [8].

In addition to the better-understood role of collecting duct principal cells in volume regulation, it is becoming increasingly apparent that the adjacent intercalated cells (ICs) also participate in regulating body fluid balance, mostly via Na^+^ and Cl^-^ transport [9–11], and through a paracrine mechanism via secreted prostaglandin E2 [12, 13]. The goal of the current study was to establish if there is any difference in the water balance between male and female mice under baseline conditions and to determine whether this involves IC function by using Atp6v1b1null mice that lack a critical subunit of the vacuolar proton-pumping ATPase (V-ATPase) that partially disrupts IC function.

## Materials and methods

### Animal experiments

V-ATPase B1 subunit-deficient (Atp6v1b1^-/-^) mice were generated, bred, and genotyped as previously described [14]. Adult (3–5 month old, 30–35 g) male and female wild-type (WT) and Atp6v1b1^-/-^ mice were housed under standard conditions and maintained on a standard rodent diet with free access to drinking water, unless otherwise indicated. All animal studies were approved by the Massachusetts General Hospital Subcommittee on Research Animal Care, in accordance with the National Institutes of Health, Department of Agriculture, and AAALAC requirements. A total of 147 animals were used to generate data shown in this article. Animals used for these experiments were bred in-house.

To assess kidney function, male and female WT and Atp6v1b1^-/-^ mice were subjected to metabolic cage monitoring [15]. Mice were placed in metabolic cages in pairs and allowed to acclimate to the cage for 4 days. Water and food intake were recorded, and total urine produced was collected under mineral oil, to avoid evaporation loss. For dehydration experiments, after an overnight (12 h) baseline (“control”) measurement, drinking water was removed from the cages for 12 h (“dehydration”) the following evening. For the urine collected under control and dehydration conditions, urine osmolality was measured using a Vapor Pressure Osmometer 5520 (Wescor, Logan, UT). Urine pH was determined using micro pH strips (pHydrion MicroFine, Micro Essential Laboratory, Brooklyn, NY) in the pH range of 5.5 to 8.0 (resolution: 0.2 pH units). Every micro pH strip was assessed by two different investigators unaware of the mouse sex, genotype, and hydration state, as described elsewhere [16]. For measuring the urine Na, K and Cl values, we used the Cornell University Animal Health Diagnostic Center facility (240 Farrier Road, Cornell University, Ithaca, NY). These values are given as mg/12 h total excretion.

All values are reported as mean ± standard deviation of the mean (SD). Statistical significance was assessed with one-way ANOVA followed by multiple comparisons in Prism 7 software (GraphPad Software, La Jolla, CA). All the actual p values are given in the figures. P < 0.05 was considered statistically significant.

### Tissue preparation

For immunofluorescence experiments, mice were anesthetized with pentobarbital sodium (50 mg/kg body weight intra peritoneal: Nembutal, Abbott Laboratories, Abbott Park, IL) and perfused through the left cardiac ventricle at a rate of 15 ml/min with PBS (0.9% NaCl in 10 mM phosphate buffer, pH 7.4) for 2 min, followed by modified paraformaldehyde-lysine-periodate fixative (PLP; 4% paraformaldehyde, 75 mM lysine-HCl, 10 mM sodium periodate, and 0.15 M sucrose, in 37.5 mM sodium phosphate) for 7–8 min, as previously described [15,17]. Both kidneys were dissected, sliced, and further fixed by immersion in modified PLP for 4 h at room temperature and subsequently overnight at 4°C, then rinsed extensively in PBS, and stored at 4°C in PBS containing 0.02% sodium azide until use.

For immunoblot analysis, mice were anesthetized and perfused with PBS for 2 min, as described above. Both kidneys were harvested, and cortical and medullary regions were separated under a microscope and then snap frozen using liquid nitrogen and stored at -80°C until use [17,18].

### Immunofluorescence

1–2 mm-thick fixed kidney slices were cryoprotected with 0.9 M sucrose in PBS overnight at 4°C, embedded in Tissue-Tek OCT compound 4583 (Sakura Finetek USA, Torrance, CA), and frozen at -20°C on a specimen disk. 5 μm sections were cut using a Leica CM3050 S cryostat (Leica Microsystems, Bannockburn, IL), collected onto Superfrost Plus charged microscope slides (Thermo Fisher Scientific, Rockford, IL), air-dried, and subjected to the immunofluorescence staining protocol as previously described [15, 19]. Briefly, the staining protocol includes tissue rehydration in PBS for 3 x 5 min, antigenic site retrieval with 1% wt/vol) sodium dodecyl sulfate (SDS) for 4 min [20], 3 x 5 min PBS washes and a 20 min incubation with Background Buster (Innovex Biosciences, Richmond, CA) to reduce nonspecific, background fluorescence. Kidney sections were subsequently incubated with an affinity-purified goat polyclonal (IgG) antibody raised against an epitope of human aquaporin-2 (AQP2) (Santa Cruz Biotechnology, Dallas, TX) diluted to 0.25 μg/ml in Dako antibody diluent (Dako, Carpinteria, CA) for 90 min and then with an Alexa 488-conjugated donkey anti-goat IgG secondary antibody (Jackson ImmunoResearch Laboratories, West Grove, PA) (14 μg/ml in Dako diluent) for 1 h, both at room temperature. Sections were rinsed in PBS for 3 x 5 min after both antibody incubations, and then mounted in SlowFade Diamond Antifade Mountant medium (Thermo Fisher Scientific, Waltham, MA). Images were acquired using a Zeiss LSM 800 airyscan confocal microscope. Images were analyzed and processed using Volocity software (Perkin Elmer, Boston, MA) and prepared using Adobe Photoshop CS6 software (Adobe Systems, San Jose, CA) and illustrations are made in GIMP software (https://www.gimp.org/) for publication.

**Fig 1:**
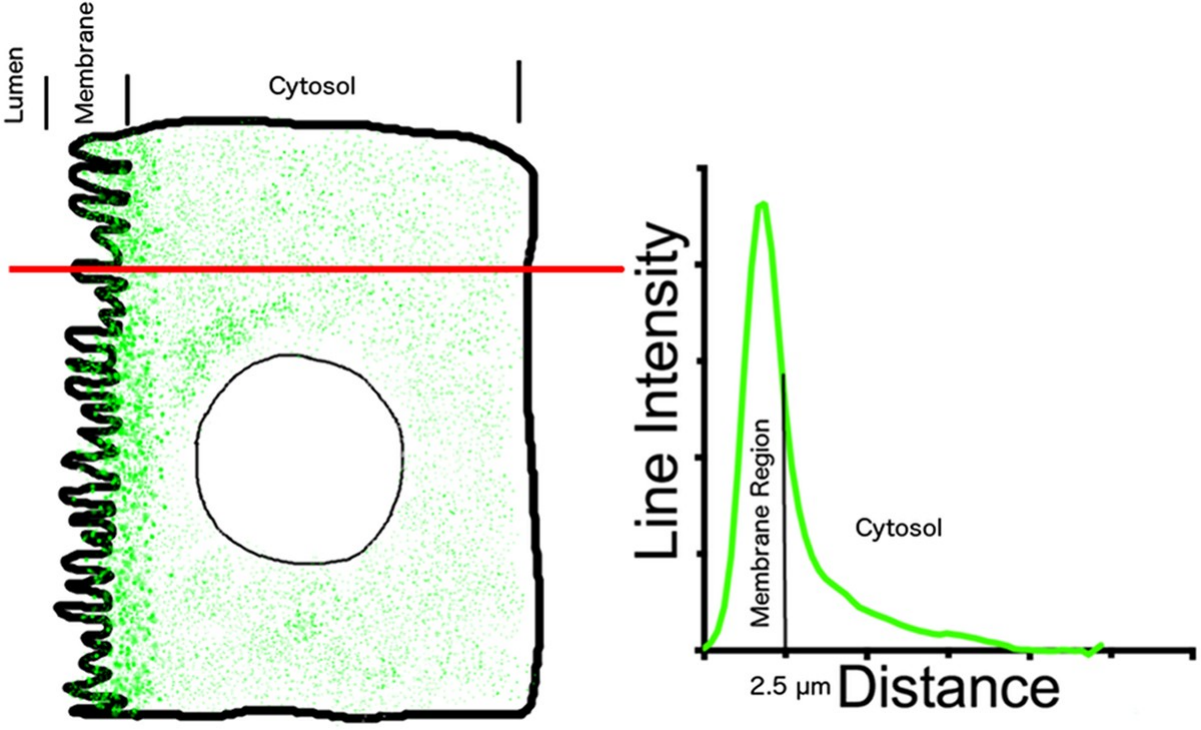
How we measured the line intensity and area under the curve. A line of 0.5 *μ*m was drawn across cells adjacent to the nucleus and pixel intensity was averaged along the line. The pixel value of the line that fell within the tubule lumen was considered as background, and the mean value from this segment of the line was subtracted from all line intensity values. The lumen-membrane junction was considered 0 distance. Total area under the curve was calculated and normalized to 100%, and percentage of area under the curve from 0 to 2.5 *μ*m was considered as the area that included the apical membrane domain. This area calculation informs us about the fraction of total AQP2 in or near the cell membrane in a given optical section.

Line intensity profiles are calculated from the average intensity value of a 0.5 μm thick line drawn across the cells, perpendicular to the apical membrane, adjacent to the nucleus. A schematic representation of the analysis is shown in Fig 1. The average value of the line in the luminal portion outside the cell was considered background, and subtracted from all intensity values along the line. The lumen—plasma membrane intersection was considered as distance 0. Line intensity values from multiple cells (minimum of 25 total cells; tissues from at least 3 animals) were averaged. In the figures in the results section, this average line intensity is shown as a black line, and the SD of the data is shown as a green area plot around the line. The normalized area under the curve from distance 0 to 2.5 μm inside the cell was considered as the intensity contribution from the apical membrane region (A_mem_).

### Immunoblot analysis

For immunoblot analysis, protein extracts were prepared from mouse kidneys as previously described [18]. Briefly, whole kidneys were cut into smaller pieces and disrupted with a PRO200 homogenizer (Pro Scientific, Oxford, CT) in 1.5 ml RIPA buffer (Boston BioProducts, Ashland, MA) supplemented with Complete protease inhibitors (Roche Applied Science, Indianapolis, IN), 1 mM EGTA and 1 mM EDTA. Homogenates were centrifuged at 4°C for 15 min at 16,200 × g, and supernatants were aliquoted and stored at -80°C. After determining total protein concentration using the Pierce Coomassie (Bradford) protein assay (ThermoFisher Scientific, Waltham, MA) with albumin as standard, 40 μg sample per lane was diluted in Laemmli reducing buffer, heated for 10 min at 90°C, loaded onto NuPAGE 4–12% Bis-Tris protein gels (ThermoFisher Scientific, Waltham, MA), and run in 3-(N-morpholino)propanesulfonic acid solution (Life Technologies, Carlsbad, CA) to separate the bands. After SDS-PAGE separation, protein was transferred to a polyvinylidene difluoride (PVDF) membrane (Bio-Rad Laboratories, Hercules, CA) and subsequently blocked (30 min) with 5% milk in Tris-buffered saline (TBS) and incubated for 1 h at room temperature with affinity-purified rabbit polyclonal antibody raised against an epitope of rat AQP2 (Alomone Labs, Jerusalem, Israel) diluted (1:5,000, 120 ng/ml) in TBS containing 0.05% Tween-20 and 2.5% non-fat dry milk. The membrane was washed for 3 x 5 min with TBS and incubated with a secondary anti-rabbit antibody conjugated to horseradish peroxidase (HRP) (Jackson ImmunoResearch Laboratories) diluted (1:5,000, 60 ng/ml) in the same buffer for 30 min at room temperature. Following three additional washes, antibody binding was visualized using the Western Lightning chemiluminescence reagent (PerkinElmer Life Sciences, Boston, MA) with Syngene G:BOX mini (Syngene, Frederick, MD). Equal protein loading was confirmed with Pierce Reversible Protein Stain kit (Thermo Scientific, Rockford, IL) for the PVDF membrane.

To quantify protein bands from immunoblot analyses, digital images of the membranes were assessed with GeneSys (Syngene, Frederick, MD). To account for sample loading variations AQP2 bands were normalized to the corresponding PVDF membrane stain obtained using the Pierce Reversible Protein Stain kit. A range of molecular weight bands was taken together to avoid sample variation.

## Results

### The response to water deprivation in wild type and V-ATPase B1 subunit deficient mice

**Fig 2:**
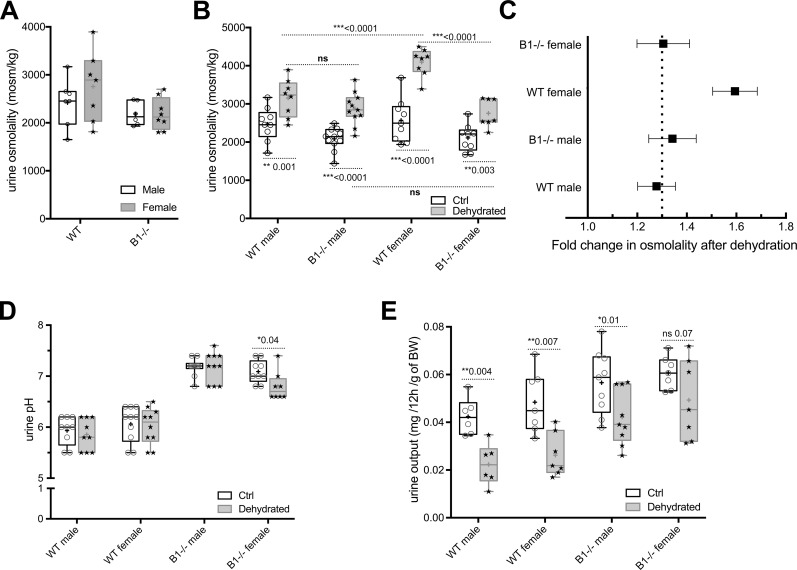
Urine measurements using metabolic cage experiments. (A) Measurements from 24 h urine showed that basal osmolality level is higher in WT female (2756 ± 734 mOsm/kg) than in male mice (2421 ± 490 mOsm/kg) and Atp6v1b1^-/-^ deficient (B1^-/-^) mice show lower osmolality values. (B) Measurements from 12 h urine of water-deprived B1^-/-^ mice. Urine osmolality after dehydration increased by 59% of the corresponding wild type value in females but by only 28% in males. In the case of B1^-/-^ animals, there was a 30% increase in females and 38% in males. (C) The concentrating ability of WT and B1^-/-^ animals is plotted for easier comparison. Dotted line represents 30% increase from baseline. (D) Under baseline conditions, urine pH is not sex-dependent and is significantly higher in B1^-/-^ than in WT animals. Dehydration significantly affected urine pH only in B1^-/-^ females. (E) 12 h water deprivation decreased urinary output by 59% in WT females and by 44% in WT males. This decrease was less in B1^-/-^ males (28%) and not statistically significant in B1^-/-^ females (29%). Under basal conditions, there was more urine production in B1^-/-^ animals compared to WT animals.

To test the baseline urine output, we adapted and collected 24 h urine from wild type (WT) and V-ATPase B1 subunit deficient (Atp6v1b1^-/-^) mice using metabolic cages. Under baseline conditions, urine osmolality trends higher in female (2,756±734 mOsm/kg, mean ± SD) than in male WT mice (2,421 ± 490 mOsm/kg). Atp6v1b1^-/-^ mice revealed no sex-dependent difference, but for both sexes urine osmolality trended towards lower values than those seen in their WT counterparts (Fig 2A).

Next, we subjected the mice to water deprivation for 12 h to assess their response to dehydration. As expected, urine osmolality increased significantly upon dehydration in all four groups studied (p≤0.003). However, water deprivation increased the urine osmolality in female WT mice by 59%, whereas in WT males it was increased by only 28%. Consequently, water-deprived WT female mice had a significantly higher urine osmolality compared to WT males (p<0.0001). This sex-dependent difference was abolished in the Atp6v1b1^-/-^ animals. Both male and female Atp6v1b1^-/-^ mice showed an approximately 30% increase in urine osmolality in response to water deprivation (Fig 2B), both similar to WT males. Accordingly, there was no statistically significant difference between water-deprived WT and Atp6v1b1^-/-^ male mice, whereas water-deprived WT females had a significantly higher urine osmolality than Atp6v1b1^-/-^ females (p<0.0001) (Fig 2B).

These sex- and genotype-dependent differences are highlighted when analyzing urine osmolality data as fold change upon water deprivation (Fig 2C). This reveals the similar increase (to approximately 1.3 fold) in urine osmolality after dehydration in WT male mice and Atp6v1b1^-/-^ mice of both sexes, whereas female WT mice exhibited a significantly greater ability to concentrate urine (~1.6 fold).

Under baseline conditions, urine pH is not sex-dependent and, as previously reported [14], it is significantly higher in Atp6v1b1^-/-^ than in WT animals. Intriguingly, water deprivation significantly affected urine pH only in Atp6v1b1^-/-^ female mice. In this group, urine pH decreased upon dehydration from 7.01 to 6.71 (p = 0.016) (Fig 1D). No statistically significant change was detected in urine pH after water deprivation in any of the other animal groups investigated.

12-h water deprivation decreased urinary output by 54% in WT female mice and by 48% in WT males (Fig 2E). This decrease was reduced in Atp6v1b1^-/-^ male mice and was not significant in Atp6v1b1^-/-^ females. These data suggest that female Atp6v1b1^-/-^ mice may lose their urine concentrating ability. This is the only group in which, while there is a trend towards lower urine osmolality values, there is no statistically significant difference between water-deprived and control animals. Individual data indicate that some of the animals in this group do respond to water deprivation by concentrating their urine, while others do not (Fig 2E). The results on urine volume are, however, less reliable than urine osmolality measurements, because the very small volumes produced by dehydrated mice can lead to measurement errors in metabolic cage studies.

Table 1 shows the urinary Na, K and Cl values. There was a general trend in the reduction of these electrolytes after water deprivation. We did not observe any sex dependent difference at baseline in both phenotypes.

**Table 1:** Effect of dehydration on 12 h urinary excretion of Na, K, and Cl.

	WT M	WT M DH	WT FM	WT FM DH	B1^-/-^ M	B1^-/-^ M DH	B1^-/-^ FM	B1^-/-^ FM DH
Na	3.3±1	2.2±0.87*	2.89±0.9	2.19±0.77*	4.1±1.4	3.24±0.8	3.6±1	3±0.98
K	18.53±6.6	13.11±5.8	19.21±3.9	10.3±2.7*^$^	19.5±6.2	16.4±3.6	18.26±3.8	12.83±4.5
Cl	8.26±2.7	5.42±2.1	8.19±1.9	4.8±1.4*	9.52±3.3	7.88±1.8	8.33±2.3	6.0±1.9

M–male, FM–Female, DH–Dehydrated. Values are expressed as mean ± SD (mg/12h), n = 6 for every data point. One-way ANOVA test followed by Tukey’s correction for multiple comparisons was conducted for statistical analysis.

*P < 0.05 compared to B1^-/-^ M

^$^P < 0.05 compared to WT FM.

### AQP2 expression in wild type and V-ATPase B1 deficient mice

**Fig 3:**
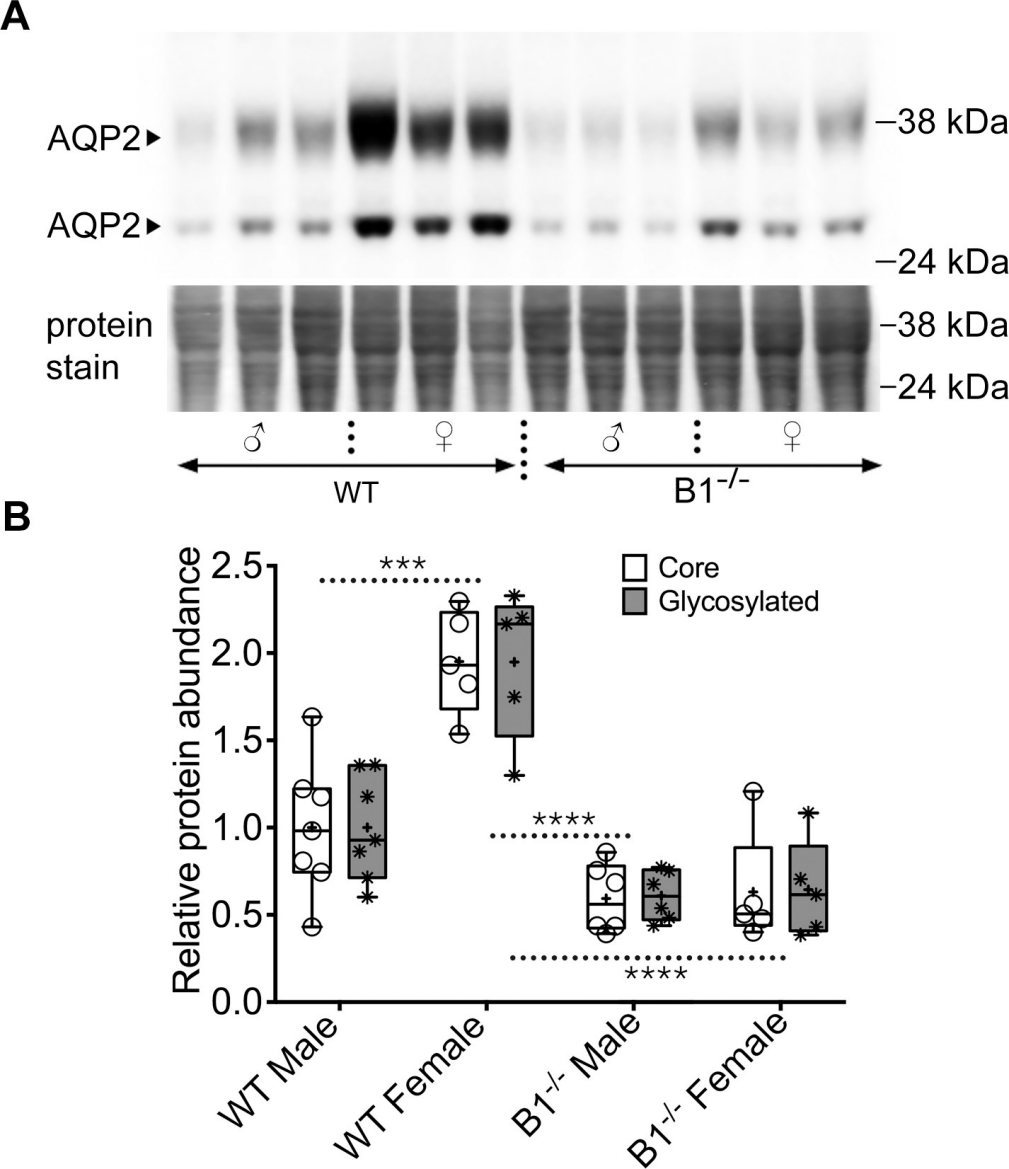
Total AQP2 protein expression in male and female WT and Atp6v1b1-/- animals. (A) Immunoblot showing the two characteristic, core and glycosylated, bands of AQP2 in the top panel. The bottom panel shows the loading control using total protein stain with the Pierce Reversible Protein Stain kit. (B) Lane intensity quantification normalized to the protein loading control shows that at baseline, WT females had the highest total AQP2 and deficiency in B1 subunit of the V-ATPase reduces total AQP2, especially in the female animals. There was also a reduction in total protein in the B1^-/-^ male animals compared to their WT counterparts, though within our tested experimental conditions it was not statistically significant. Deficiency in the B1 subunit in female animals reduced the total protein content to such an extent that it became comparable to the level of protein in male animals of both genotypes.

Immunoblotting analysis was used to assess total AQP2 protein levels in the cortex and medulla of male and female WT and Atp6v1b1^-/-^ mice. Significant sex- and genotype-specific differences were detected in AQP2 protein abundance in the cortical region (Fig 3). Female WT mice show significantly higher AQP2 protein expression compared to their male counterparts. In the Atp6v1b1^-/-^ mice, females show a significant decrease in AQP2 compared to female WT animals, whereas in male animals, the apparent reduction in AQP2 expression compared to their WT counterparts is not statistically significant. Genetic deletion of Atp6v1b1 eliminated the sex dependent difference in AQP2 protein levels (Fig 3, S1–S3 Figs). Glycosylated and non-glycosylated bands of AQP2 showed a similar expression trend in all four groups tested.

### AQP2 subcellular localization in wild type and V-ATPase B1 deficient mice

Given the critical role played by the water channel protein aquaporin-2 (AQP2) in maintaining water balance [21–25], we used immunofluorescence staining to investigate the subcellular localization of AQP2 in collecting duct (CD) principal cells (PCs) in control and water-deprived male and female WT and Atp6v1b1^-/-^ mice. Our immunostaining data indicate that under control conditions AQP2 localizes mostly to the cytosolic and subapical domains of PCs as expected. Upon 12 h water deprivation, AQP2 exhibits a tendency to relocate more tightly towards the apical pole of PCs where it mediates water reabsorption from the tubule lumen.

**Fig 4:**
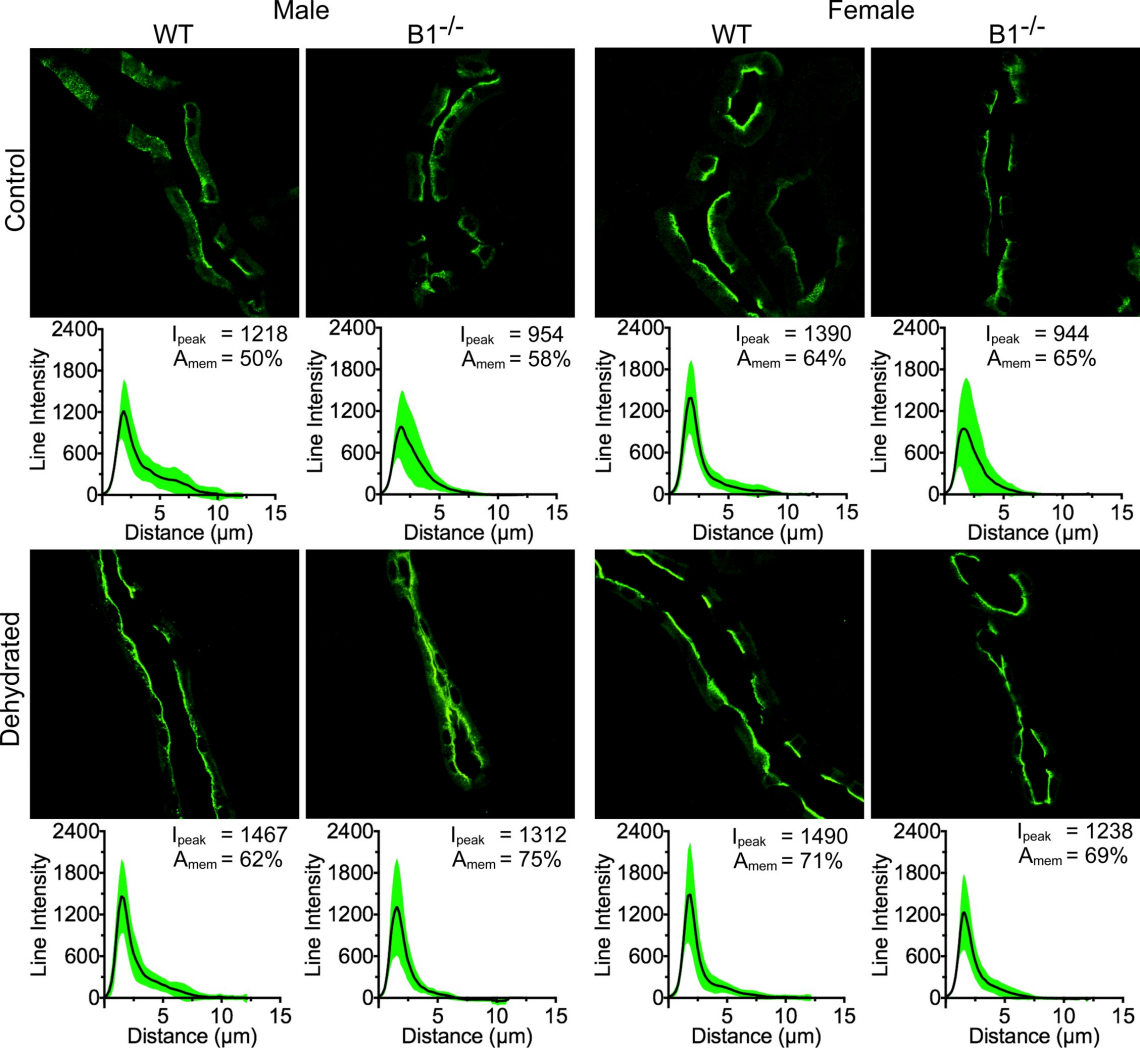
Male animals show the highest level of AQP2 regulation upon dehydration in the cortical collecting duct. Immunofluorescence imaging showing the distribution of AQP2 in the kidney principal cells (PCs). Under baseline conditions, AQP2 localization in the cortical collecting duct (CCD) PCs is sex-independent in both WT and Atp6v1b1-/- (B1^-/-^) mice. Upon dehydration AQP2 localization partially shifted towards the apical membrane. This effect was more pronounced in the male animals, of which B1^-/-^ mice showed the strongest response. Every panel has an average line intensity plot (black line) showing the distribution of protein within the cell. SD of the data is shown as a green area plot around the mean value plot. Peak intensity (I_peak_) values and the total intensity distribution near the plasma membrane (A_mem_) are also shown.

In the case of WT animals, the shift in subcellular localization towards the PC apical membrane of cortical CDs upon dehydration was greater in male compared to female mice (24% and 10% respectively). However, we found that this localization shift was highest in the case of Atp6v1b1^-/-^ male mice (29% increase in protein localization towards the apical membrane region) and lowest (6%) in the case of Atp6v1b1^-/-^ female animals (Fig 4). The peak intensity change in the case of both female and male Atp6v1b1^-/-^ was comparable (31% female vs 37% male). Under control conditions, female WT animals showed the highest immunofluorescence intensity among all groups (Fig 4) but had the lowest “apical” relocation pattern upon dehydration.

**Fig 5:**
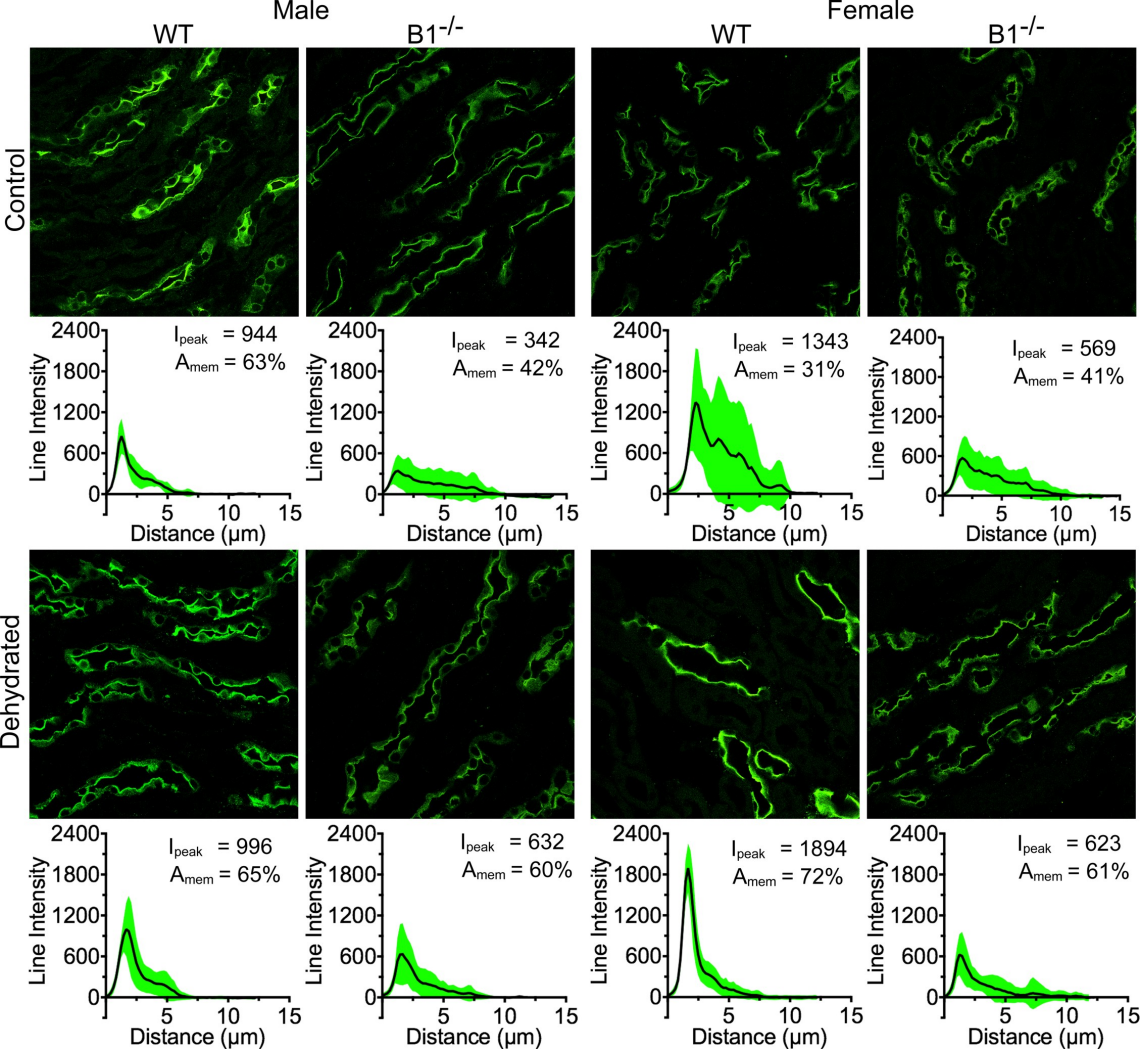
Female WT animals show the highest level of AQP2 regulation upon dehydration in the inner segment of the outer medulla. WT male animals do not show much regulation in this part of the kidney (2% increase in peak intensity and AQP2 in the membrane area, 1^st^ column), while Atp6v1b1-/- (B1^-/-^) males show a considerable amount of regulation with a 42% increase in AQP2 in the membrane region and an 84% increase in peak intensity (2^nd^ column). The highest amount of regulation was seen in the case of WT female animals with a 132% increase in the AQP dependent fluorescence in the membrane region and a 41% increase in peak intensity (3^rd^ column). Whereas female B1^-/-^ animals showed a mere 9% increase in peak intensity, there was a 48% increase in AQP presence in the membrane region (4^th^ column).

The subcellular localization of AQP2 in the outer stripe (OS) of the outer medulla (OM) was predominantly in the apical domain under control conditions and water deprivation did not markedly alter this localization (data not shown) either in WT or Atp6v1b1^-/-^ mice. In the inner stripe (IS) of the OM of WT male mice, AQP2 localizes predominantly to the apical plasma membrane under control conditions and water deprivation did not further mobilize AQP2 protein towards the membrane. In contrast, we found a huge variability in cellular AQP2 distribution in the WT female animals in this region of the kidney. Some female animals were similar to WT male animals in AQP2 distribution, while in some others it was much more cytosolic. However, after water deprivation, we quantified a large shift in distribution of AQP2 towards the plasma membrane. This relocation was the most striking among all regions and phenotypes we studied (Fig 5). There was an increase of 132% in AQP2 in the apical membrane area and a 41% increase in peak intensity in these female mice. In contrast to this significant sex difference in WT mice, both male and female Atp6v1b1^-/-^ animals showed a similar shift in AQP2 subcellular localization after dehydration (43% and 49% respectively, Fig 5).

**Fig 6:**
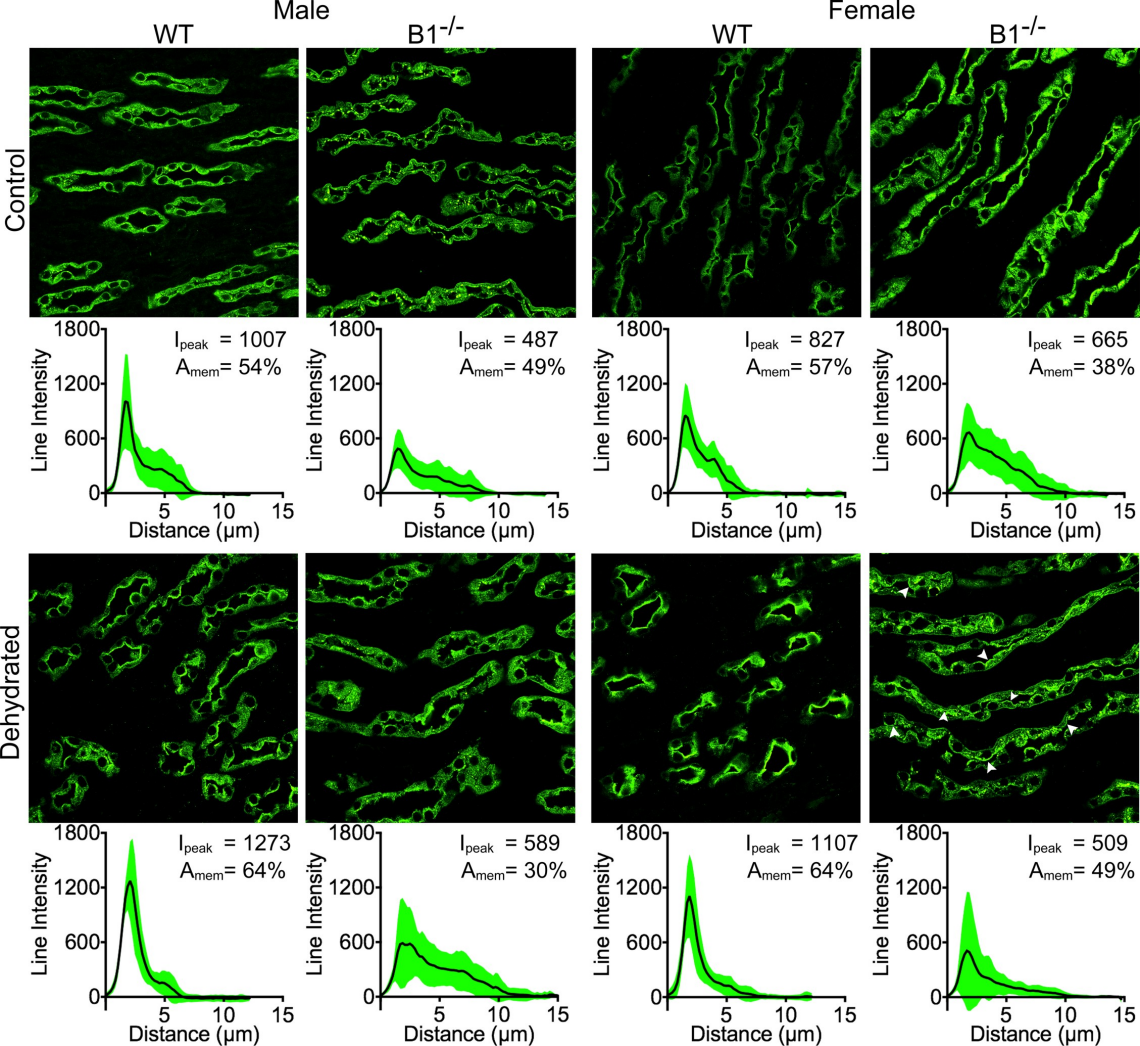
Atp6v1b1^-/-^ animals show a negligible amount of regulation in the base of the inner medulla. Male WT animals showed a 26% increase in the peak intensity (1^st^ column) and female WT showed a 34% increase in peak intensity (3^rd^ column). In the case of B1^-/-^ males, there was actually a reduction in AQP2 in the membrane region, but the peak intensity increased (2^nd^ column). In the case of female B1^-/-^ animals, there was a considerable reduction in peak intensity (30%), though the protein in the membrane region also increased (29%). In this case, even though the average peak intensity was reduced, there are many cells that had a very clear membrane expression of AQP2 (white arrowheads), indicating a heterogeneous cell regulation of AQP2 (4^th^ column).

At the base of the IM, i.e. in the region of the IM that still contains intercalated cells (ICs), we saw a similar response to water deprivation in WT male and female mice (Fig 6, 1^st^ and 3^rd^ column). In the case of male Atp6v1b1^-/-^ mice, water deprivation failed to mobilize AQP2 towards the plasma membrane (decreased by 63%), but there was a 20% increase in the peak intensity of AQP2 staining (Fig 6, 2^nd^ column). In female Atp6v1b1^-/-^ mice, even though the overall response in mobilizing AQP2 after dehydration is similar to WT animals, the peak average intensity was actually reduced by 30% (Fig 6, 4^th^ column). Interestingly, we observed some cells with a very tight apical plasma membrane localization of AQP2 (white arrowheads), indicating a quite heterogeneous cellular response to water deprivation (Fig 6). While averaging the line intensity of fluorescence from different cells reduced the average peak intensity, the cells with a marked apical membrane expression in this region probably play an active role in water reabsorption. Also from the distribution of SD in the plot (bottom most plot in the 4^th^ column) it is apparent that there are some cells with high intensity and a clear peak of apical AQP2 staining.

**Fig 7:**
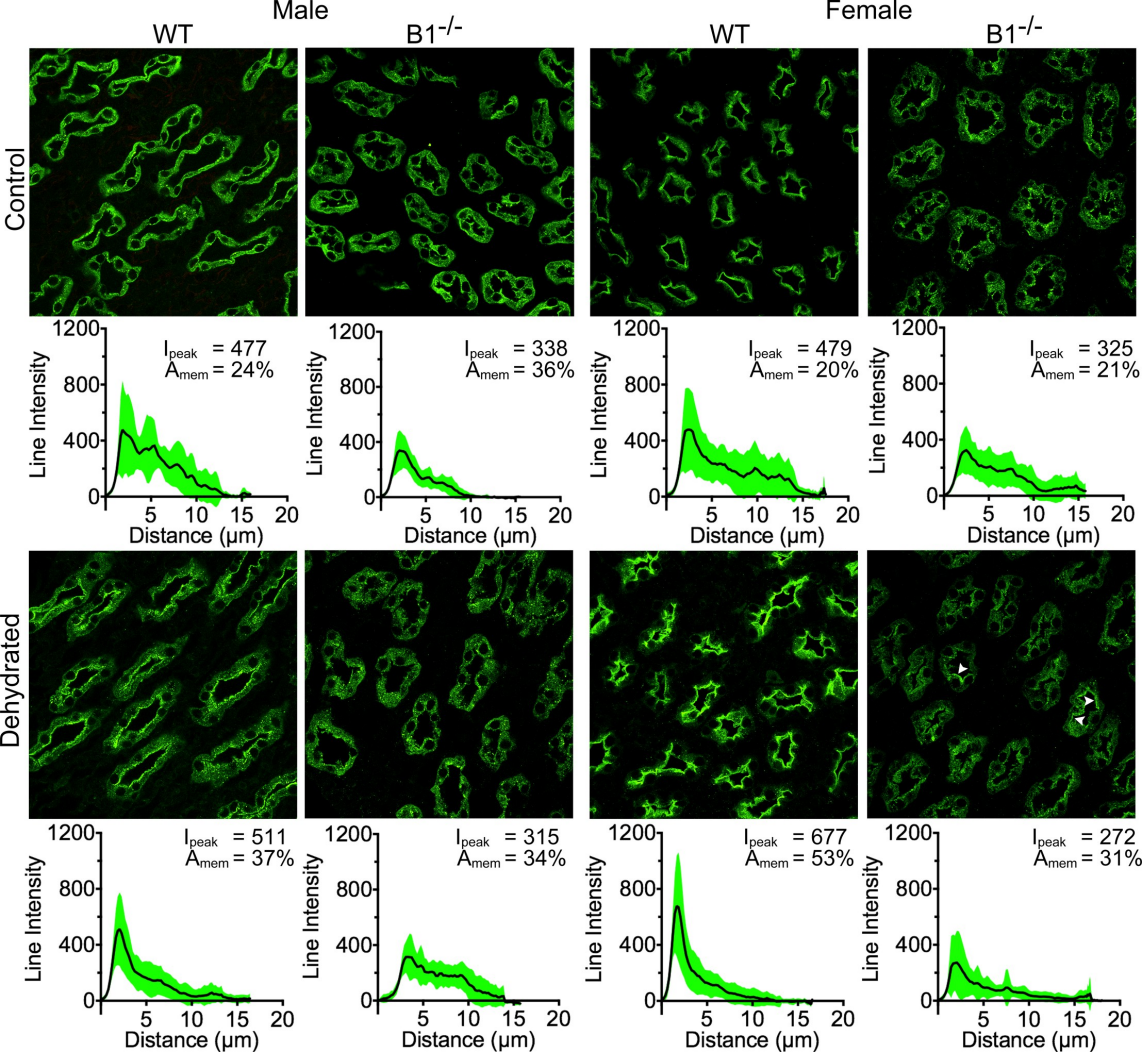
Female WT mice regulate AQP2 more than males in the tip of the inner medulla. Male WT animals showed little increase of peak intensity after dehydration (only 7%), even though the presence of AQP2 in the membrane region increased by 54% (1^st^ column). On the other hand male B1^-/-^ mice did not exhibit any regulation of AQP2 upon dehydration (2^nd^ column). In contrast, female WT animals had a considerable amount of regulation of AQP2 in this region with a 41% increase in peak intensity and a 160% increase in AQP2 in the membrane region (3^rd^ column). Female B1^-/-^ animals also showed an increase in AQP2 in the membrane vicinity, but peak intensity was reduced (4^th^ column). Similar to the base of IM, there were many cells with a clear membrane expression of AQP2 (white arrowheads) although the overall peak intensity was reduced.

At the tip of the IM, which consists entirely of PCs, the overall AQP2 fluorescence intensity in Atp6v1b1^-/-^ mice was less than in their WT counterparts. AQP2 responds well to water deprivation in male and female WT mice, by moving from a largely cytosolic distribution to a more apically polarized localization (Fig 7). Compared to WT males (54% increase in AQP2 in the vicinity of the apical membrane, and a 7% increase in peak intensity), AQP2 subcellular distribution is affected to a much greater extent by water deprivation in WT female mice (265% increase in AQP2 close to the apical membrane, and a 41% increase in intensity). The tip of the inner medulla in the WT female animals shows a response very similar to the inner stripe, but the IS had nearly 3 times greater peak intensity values. Again, this response is visibly completely blunted in Atp6v1b1^-/-^ males, where no PCs clearly expressing AQP2 on their apical membranes could be detected (Fig 7). In contrast, female Atp6v1b1^-/-^ animals had a 48% increase in the AQP2 fluorescence in the apical membrane region, which is 5.5 times less than that mobilized by WT females (265% increase). In addition, there was a reduction in average peak intensity in the Atp6v1b1^-/-^ females. Similar to the base of the IM, in female Atp6v1b1^-/-^ animals there were some cells in the tip of the IM with a very strong apical membrane expression of AQP2 (white arrowheads), indicating a stronger, but heterogeneous response in female mice compared to Atp6v1b1^-/-^ males. In Atp6v1b1^-/-^ female animals, compared to the region at the base of the IM, the percentage of PCs in which AQP2 translocated to the cell plasma membrane was reduced, suggesting that in the tip region of the IM, PCs are partially precluded from responding to the dehydration challenge. Taken together, all of these significant differences in AQP2 relocation after dehydration would contribute to the apparent concentrating advantage of female WT animals over their male counterparts, and to the loss of this sex difference in the Atp6v1b1^-/-^ female mice.

To ensure that the differences seen in urine concentrating ability depend on AQP2 expression and membrane localization only, we also quantified the percentage of PCs and ICs in the IS and base of IM CD in the four animal groups. This quantification was performed from immunofluorescence images using AQP2 as a PC marker and V-ATPase as an IC marker as previously described [17]. Four mice from each animal group (male and female WTs and male and female Atp6v1b1^-/-^ mice) were included in the quantification, and an average of over 1,650 total cells per animal were counted. Our data indicate that the ratio between PCs and ICs does not undergo significant sex-dependent changes in the two mouse strains: in WT male mice 69.1 ± 1.9% of total CD cells were PCs, whereas in WT females PCs accounted for 69.4 ± 1.0% of cells. The V-ATPase B1 subunit deficiency did not induce any significant changes in the PC:IC ratio either, as 68.4 ± 1.5% of total cells in female Atp6v1b1^-/-^ mice were PCs, compared to 69.4±1.0% in their WT counterparts. The lowest percentage of PCs was found in Atp6v1b1^-/-^ males (66.8 ± 0.9%), which was not significantly different when compared with either WT males or Atp6v1b1^-/-^ female mice.

## Discussion

Men tend to hydrate more than women during prolonged exercise [1] but females are at increased risk for exercise-associated hyponatremia (EAH), caused by hypervolemia [2,3,5,26]. While drinking patterns may partially explain these observations, important regulators of the urinary concentrating mechanism are likely to be involved. In particular, the role of the collecting duct water channel AQP2 in this sex-dependent difference is unknown. Here we show that that female mice concentrate urine better than their male WT animals upon dehydration, and that AQP2 is differentially expressed and regulated in a sex-dependent manner in the kidney. Interestingly, this marked sex difference is completely absent in mice lacking the B1 subunit of the V-ATPase in the renal intercalated cells, whose emerging role in ion and fluid homeostasis [13]—in addition to acid-base balance—is supported by our data.

This prompted us to investigate the water balance differences in male and female Atp6v1b1^-/-^ mice in comparison with WT animals. There was no statistical difference in urine output and concentration between male and female mice of either genotype under control conditions, but urine volume was significantly greater in both male and female Atp6v1b1^-/-^ mice compared to WT controls. The greater urine volume was accompanied by a trend towards lower osmolality, but this was highly variable and did not reach statistical significance. This increased urine output in Atp6v1b1^-/-^ mice has been reported previously but potential effects of the sex of the mice studied were not reported [13, 14]. Despite the similar results under baseline conditions between male and female mice, our overnight water deprivation study revealed that female WT animals were able to concentrate urine remarkably better than their male counterparts (59% vs ~27% increase in urine osmolality). Overnight water deprivation was chosen because we found that nearly 90% of the urinary excretion occurred during the night in these animals. As expected, this dehydration protocol reduced urine volume in all mice although due to a larger variability, the volume reduction in female Atp6v1b1^-/-^ fell short of achieving statistical significance. As mentioned above, urine volumes are very small after dehydration in mice, and are subject to experimental errors due to collection problems. Therefore, we consider urine osmolality to be a more reliable indicator of concentrating ability in dehydrated mice. In absolute values of osmolality difference, female mice increased urine osmolality by 1,526 mOsm/kg from baseline after dehydration, compared to a 686 mOsm/kg increase for male mice. Unexpectedly, this 2.2 fold difference between the sexes was lost in Atp6v1b1^-/-^ mice, and female animals of this genotype actually concentrated less than their male counterparts after dehydration (0.8 fold: 644 mOsm/kg in females vs. 796 mOsm/kg in males). In other words, knocking out the B1 subunit of the V-ATPase from the renal collecting duct intercalated cells impaired the ability of Atp6v1b1^-/-^ female mice to concentrate their urine compared to WT females. The difference between dehydrated females and controls of the two genotypes was 1,526 vs. 644 mOsm/kg. In contrast, even though the baseline urine osmolality in male Atp6v1b1^-/-^ mice was lower than in their WT counterparts, their ability to concentrate urine is not modified by knocking out the B1 V-ATPase subunit (difference between dehydrated and control: 686 vs. 796 mOsm/kg). In summary, knocking out the V-ATPase B1 subunit, which adversely affects the function of renal intercalated cells, results in elimination of a sex-specific “advantage” in concentrating ability after dehydration that is shown by WT female mice.

Because of the lower activity of A-intercalated cells, Atp6v1b1^-/-^ animals have less acidic urine compared to WT animals as previously reported [14]. Dehydration did not affect urine pH values in WT mice, but the female Atp6v1b1^-/-^ animals had a significantly lower urine pH than B1 deficient males and non-dehydrated B1 deficient females. These data suggest that urinary acidification is less impaired in Atp6v1b1^-/-^ females compared to males after dehydration. While this is possibly related to a less disrupted function of intercalated cell mediated protein secretion in female B1 null mice, there are many other factors that could account for this pH difference, including the availability of urinary buffers such as NH_3_ and PO_4_^—^, as well as the activity of other acid/base transporting proteins along the urinary tubule. These issues remain to be examined.

One of the membrane proteins that is critical for urine concentration is AQP2. A higher expression level of AQP2 was found in the WT female animals compared to males, both in the cortex and the medulla. However, this striking sex-dependent difference is lost in the Atp6v1b1^-/-^ animals. Females of this genotype had much lower levels of AQP2 that were not different from WT or Atp6v1b1^-/-^ males. This result is consistent with the functional data that we obtained, and shows that knocking out the B1 subunit of the V-ATPase affects AQP2 expression levels and, consequently, may explain their reduced ability to concentrate urine upon dehydration compared to WT females. In addition to the degree of AQP2 expression, AQP2 trafficking is an important mechanism that regulates water reabsorption in the collecting duct. Upon dehydration, vasopressin released into the circulation acts via the V2R to cause apical membrane accumulation of AQP2 in principal cells [21–25]. This shift in cellular localization from cytoplasmic vesicles to the plasma membrane results in osmotic water movement across principal cells into the renal interstitium, which concentrates the luminal fluid. In the cortical collecting duct, male animals showed the strongest shift in AQP2 fluorescence intensity towards the apical membrane region upon dehydration, and Atp6v1b1^-/-^ males showed the largest response. In the female animals this difference upon dehydration was low; in the case of Atp6v1b1^-/-^ animals, there was only a 4% increase in AQP2 intensity in the membrane region after dehydration. On the other hand, male Atp6v1b1^-/-^ mice showed a 17% increase in fluorescence intensity in the membrane region. These data in the cortex were unexpected, but indicate that much of the increased urine concentrating ability in WT female animals probably occurs in the medullary region.

Indeed among different medullary regions studied in the WT animals, female animals had the highest peak AQP2 intensity before and after dehydration, except in the base of the IM. The strongest response to dehydration of all studied groups, including peak intensity and mobilizing the AQP2 to the apical membrane, was observed in the IS region in female WT animals. The clear and strong apical localization of AQP2 in female animals after dehydration, together with the higher total protein expression, can explain the increase in urine concentrating ability seen in the female animals. The lack of comparable AQP2 regulation, especially in the IS, and a lower AQP2 expression in the Atp6v1b1^-/-^ female animals might be one reason why these animals lose the ability to concentrate the urine as well as the WT females, and resemble male animals in their concentrating ability. In addition, the relatively low quantity of total AQP2 and lack of regulation in the IM might be a reason why male Atp6v1b1^-/-^ mice show a lower total urine osmolality than to their WT counterparts.

Gueutin et al. [13] attributed the difference in water balance in the Atp6v1b1^-/-^ animals compared to WT mice to the increased secretion of prostaglandin E2 (PGE2) from the cortical collecting duct B-type intercalated cells. Prostaglandin is known to regulate water and Na balance in the kidney [12,13,27] and its production and secretion are also sex dependent [28,29]. With the current knowledge it is difficult to attribute the higher prostaglandin level in one sex to reduced water uptake, as there are many other intermediate players taking part in water homeostasis. Prostaglandin in the renal cortex of female rats is higher, but male animals have a much greater amount of OAT-PG, a prostaglandin specific transporter, and it is strongly regulated by testosterone [29]. Furthermore, in Sprague-Dawley rats, V2R protein and mRNA levels are higher in females compared to males [8] and the antidiuretic response to vasopressin (VP) is also higher in female than in male rats [30]. Moreover, hypertonic saline infusion experiments in humans showed that females and males clear water similarly at rest, but females have lower VP levels in the blood, suggesting a higher renal sensitivity to vasopressin [31]. The greater renal sensitivity in females could also arise from the higher V2R expression.

Finally, how might these observations be related to exercise-associated hyponatremia (EAH) in human females? In most clinical situations, excess water retention is the reason behind EAH [1–3,26,32]. The effect of any of sex hormones on concentrating ability remains to be explored, but infusing sex hormones did not affect the serum Na^+^ levels in laboratory studies on EAH in human females [32]. The difference in AQP2 expression levels and AQP2 membrane accumulation after dehydration between females and males may, however, be a contributing factor. While under some conditions, the ability to conserve water would be an advantage, females may be less able to produce sufficient dilute urine to shed a water load–particularly after rapid or inappropriate attempts to rehydrate after exercise.

**Fig 8:**
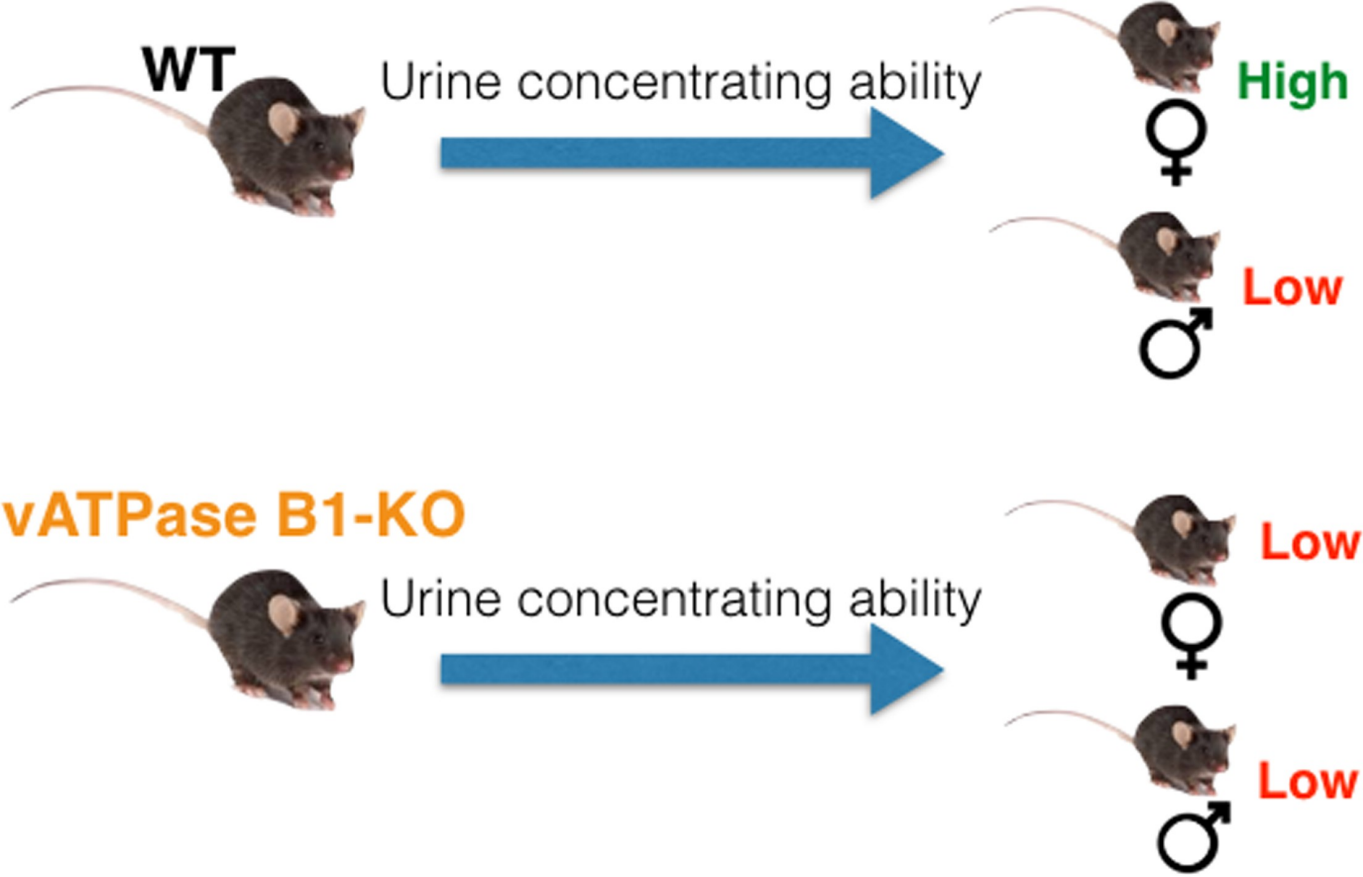
Schema depicting the overall difference in water regulation in the Atp6v1b1^-/-^ mice compared to WT. Ablation of the V-ATPase B1 subunit decreases the urine concentrating ability of mice both under baseline conditions and after dehydration. Loss of the B1 subunit eliminates the apparent advantage in water concentrating ability of female animals over the male animals that was found in WT mice.

In conclusion, our study shows that female mice concentrate urine better than males when deprived of water and this “advantage” is abolished in the animals that are deficient for the B1 subunit of the V-ATPase in the intercalated cells (Fig 8). While the involvement of intercalated cells in body fluid homeostasis is increasingly recognized, the crosstalk between V-ATPase function in these cells and AQP2 expression and trafficking in adjacent principal cells remains to be elucidated in future studies.

## Supporting information

S1 Fig Original western blot from Fig 3A. Western blot image of whole PVDF membrane overlapped with molecular weight marker is displayed.
(TIF)

S2 Fig Original western blot from Fig 3A. Image of the whole PVDF membrane showing the western blot depicted in Fig 3 is shown.
(TIF)

S3 Fig Membrane stain for western blot loading control from Fig 3A. Image of total protein stain using Pierce Reversible Protein Stain kit of the entire PVDF membrane is shown as loading control.
(TIF)
