# Supplementary figures and images for: Sex-dependent differences in water homeostasis in wild-type and V-ATPase B1-subunit deficient mice
Source: PLoS One. 2019 Aug 6;14(8):e0219940. doi: 10.1371/journal.pone.0219940 (PMC6684071; doi:10.1371/journal.pone.0219940)

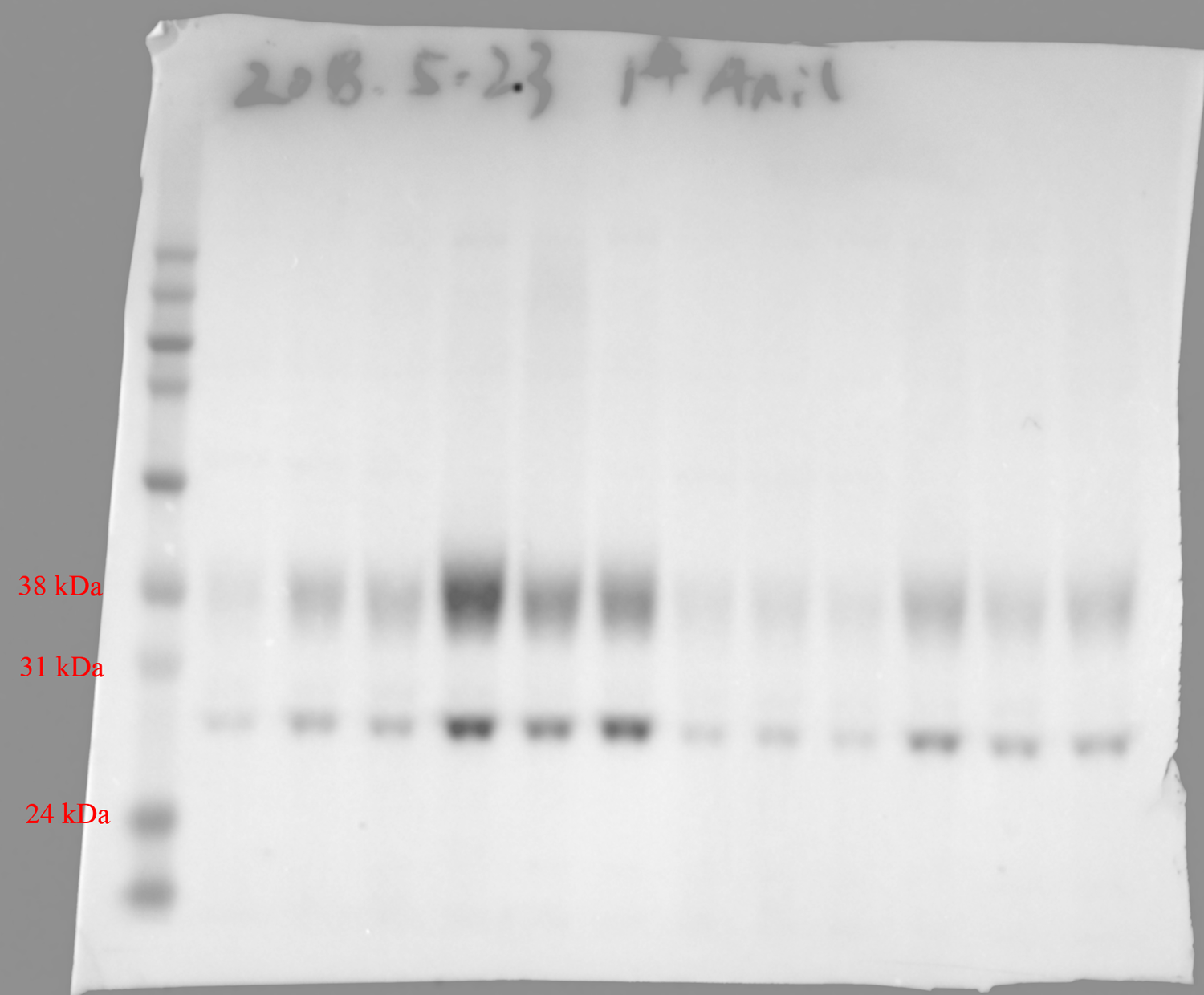

Supplement: S1 Fig — Western blot image of whole PVDF membrane overlapped with molecular weight marker is displayed. (TIF) [file pone.0219940.s001.tif]

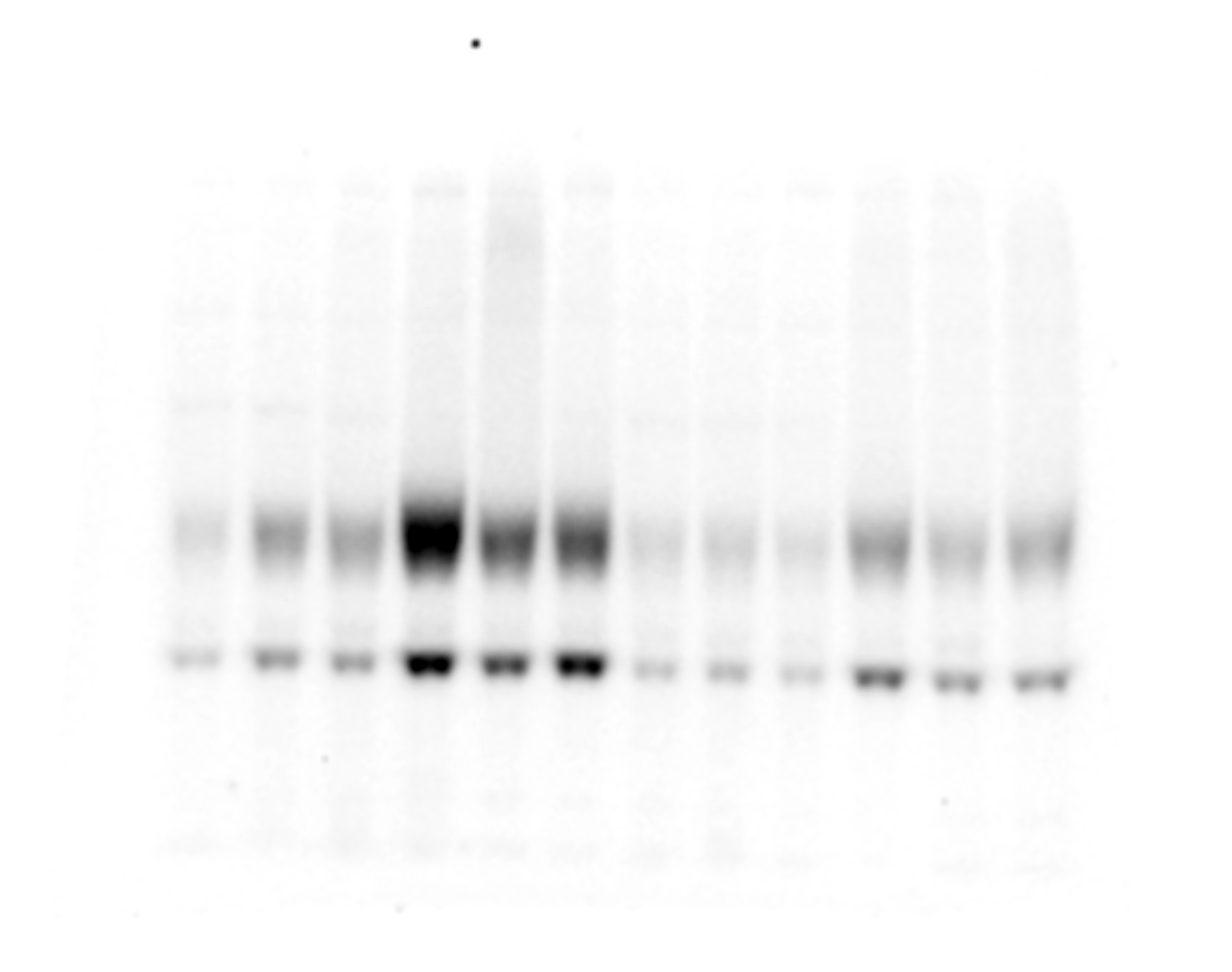

Supplement: S2 Fig — Image of the whole PVDF membrane showing the western blot depicted in Fig 3 is shown. (TIF) [file pone.0219940.s002.tif]

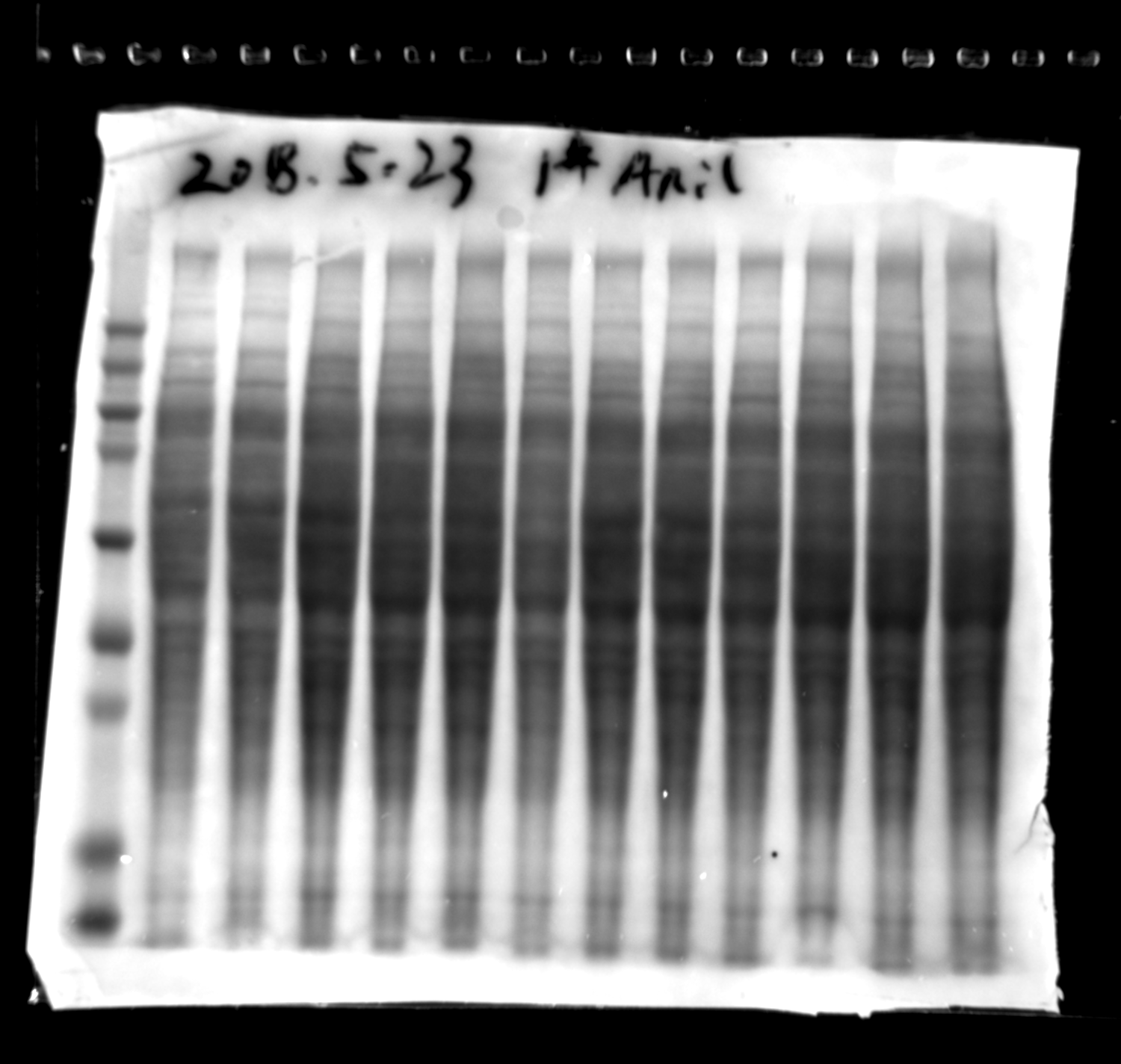

Supplement: S3 Fig — Image of total protein stain using Pierce Reversible Protein Stain kit of the entire PVDF membrane is shown as loading control. (TIF) [file pone.0219940.s003.tif]
